# Comparison of Three Circular Frames in Lower Limb Deformity Correction: A Biomechanical Study

**DOI:** 10.7759/cureus.25271

**Published:** 2022-05-24

**Authors:** Kenan Basha, Ahmad Alawadhi, Maha Alyammahi, Mohamed Sukeik, Hayder S Abdulhadi, Ajay P Dsouza, Ibrar Majid, Sattar Alshryda

**Affiliations:** 1 Orthopaedics and Traumatology, Mohammed Bin Rashid University of Medicine and Health Sciences, Dubai, ARE; 2 Orthopedics and Traumatology, Mohammed Bin Rashid University of Medicine and Health Sciences, Dubai, ARE; 3 Trauma and Orthopaedics, Rashid Hospital, Dubai, ARE; 4 Orthopaedics, Dr. Sulaiman Al Habib Hospital, Dammam, SAU; 5 Orthopaedics, Rashid Hospital, Dubai, ARE; 6 Radiology, Al Jalila Children's Speciality Hospital, Dubai, ARE; 7 Pediatric Orthopaedics, Al Jalila Children's Speciality Hospital, Dubai, ARE; 8 Pediatric Orthopaedics and Trauma, Al Jalila Children's Speciality Hospital, Dubai, ARE

**Keywords:** femur, tibia, orthex frame, truelok hexapod, taylor spatial frame, deformity correction, lower extremity deformities

## Abstract

Background

The use of circular frames in correcting lower limb deformity is well-established and has evolved dramatically over the years. Three new frames have been introduced recently, and this study is set to compare them in terms of accuracy and efficiency in correcting a similar long bone deformity. These frames are the Taylor Spatial Frame (TSF; Smith & Nephew, London, United Kingdom), the Truelok Hexapod System (TL-HEX; Orthofix, Lewisville, Texas), and Orthex (OrthoPediatrics, Warsaw, Indiana).

Methods

This is a biomechanical study comparing the above three types of circular frames to correct similar deformities in Sawbones models. The deformities that are compared were: (1) 30° valgus deformity of the distal femur; (2) 30° varus deformity of the proximal tibia.

Each frame was applied to the deformed bone in the standard way that we apply to normal bone. X-rays were taken before and after the deformity correction. The frames’ software was used to estimate the deformities. The variations between the software’s estimations and the known bone deformities were compared. Residual deformity after initial correction and the number of re-programmings was compared among these three frames. The least residual deformity and re-programming is the favorable outcome.

Results

All the Sawbones models had a 30° actual coronal angulation. The Orthex software estimated the deformity at around 25.35° (SD 4.6), TSF 25.6° (SD 2), and TL-HEX 29.87° (SD 2.1). One-way analysis of variance (ANOVA) showed a significant difference in the findings (P-value 0.014).

Accuracy was measured by comparing the residual deformity in angulation in the coronal plane after the first and second correction. The Orthex median residual deformity was 1°, TSF was 2.5°, and TL-HEX was 3° with a range of less than 5° for all of them. The independent samples Kruskal-Wallis test shows that there is no significant difference between the three groups (P=0.549).

The frequency of strut changes required throughout the correction was not significant among the three frames using the Fisher exact test (P=0.336). TSF struts are not designed to be readjusted.

Conclusion

The three frames were comparable in terms of accurate correction of the two deformities, strut changes, and strut adjustments. The TL-HEX frame software was superior to other frames in terms of analyzing the deformity but the difference, although statistically significant clinically, was not.

## Introduction

Bone deformity is defined as structural deviation or distortion of the bone’s shape from its normal alignment, length, or size. This can be caused by several congenital or acquired conditions, including metabolic bone disorders, such as rickets and osteomalacia; genetic disorders, such as osteogenesis imperfect and neurofibromatosis; neuromuscular conditions, such as cerebral palsy and Charcot-Marie-Tooth disease; skeletal dysplasia such as achondroplasia; vascular malformation; tumors such as bony exostosis; congenital anomalies such as fibular and tibial hemimelia and trauma [1]. Trauma is by far the most common cause of bone deformity, and it is arguably the easiest to treat. Congenital bone deformity, in contrast, is more complex and is almost always associated with soft tissue contractures that require gradual correction [2]. Bone deformity is described as angulation, translation, or both in each of the three planes of geometry, producing a matrix of deformity as shown in Appendix 1.

Our understanding of the assessment and management of deformity has progressed a lot in the last century. Professor Gavriil Ilizarov (1921-1992) from the former Soviet Union is widely regarded as the father of modern deformity correction [3]. He used circular frames to treat non-united fractures by compressing the two fragments of broken bones [4-7].

Dr. Charles Taylor, an orthopedic surgeon from Memphis, advanced the Ilizarov frame by connecting the two rings with six telescopic struts (instead of the four threaded rods) that can be independently lengthened or shortened to bring the two rings to any desired position. Furthermore, he developed a computerized way to correct deformities by combining several engineering principles. The frame was named the Taylor Spatial Frame (TSF) and was made by Smith & Nephew (London, United Kingdom) under the patency law agreement (Appendix 2).

Taylor Spatial Frame (TSF) was introduced in clinical practice in 1994. It was used in trauma and deformity correction settings with great success. One study showed 91% of fractures had complete union with no further surgeries required in a study of 57 femoral and tibial fractures undergoing fixation with the TSF [8]. Eighty-nine procedures were performed in children with congenital deformities and 39 in children with acquired deformities using TSF and there was a satisfactory correction in all but three patients who needed further surgeries [9].

One biomechanical study compared the TSF and the Ilizarov frame and found that the TSF is superior to the Ilizarov frame in terms of mechanical properties but not in terms of correction accuracy [10].

The patency of TSF expired three years ago, opening the door for three more devices to be introduced to clinical practice, namely, the TrueLok Hexapod (TL-HEX) by Orthofix (Lewisville, Texas), Orthex by OrthoPediatric (Warsaw, Indiana), and the Multi-Axial correction system (MAX frame) by Depuy Synthes (Warsaw, Indiana).

In order for these frames to get a market share from the TSF, they have to come up with an innovation that TSF does not have. Appendix 3 summarizes some of these innovations and differences among these frames, as they have implications for our findings.

One important concept in frame deformity correction using computerized methods is the proximal and distal referencing concept. Proximal referencing means using the proximal ring as a reference point for measuring deformity and the frame mounting parameters, which are important for the software to produce the prescription for deformity correction. Distal referencing refers to using the distal ring instead of the proximal ring to achieve the same. While the aim is to use the proximal referencing as it is intuitive, there are a few occasions when distal referencing is preferable. Depending on the type of the frame, with distal referencing, the surgeons may need to add or change some data to overcome the limitations. For example, changing the medial to the lateral description or adding a certain amount of rotation to the referencing ring. The closest analog to this is holding the TV remote upside down and trying to change channels. This study is set to compare these frames while correcting bone deformities.

## Materials and methods

**Figure 1:**
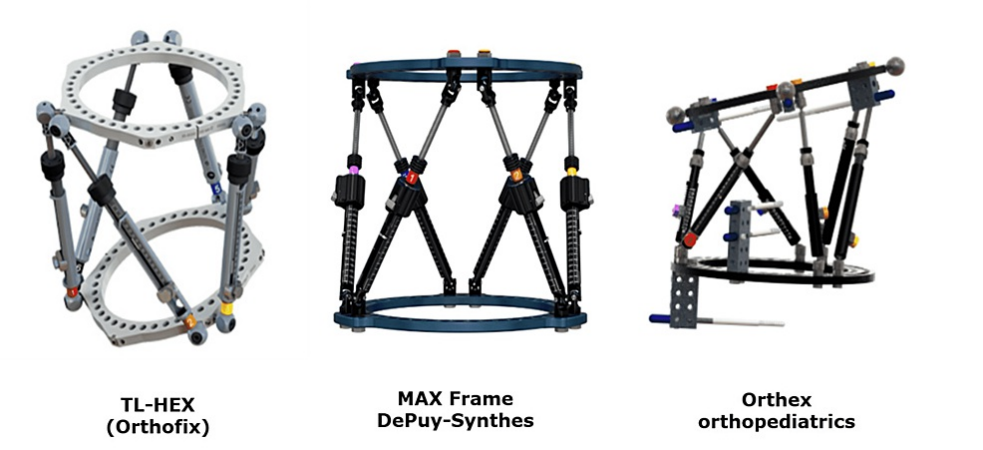
Images of the three frames have been recently introduced in clinical practice

This is a biomechanical study comparing three circular frames, namely, TSF, TL-HEX, and Orthex in terms of the accuracy of deformity estimation using the frames’ software, the precision of initial correction using the parameters of residual deformity after correction, the frequency of re-programming to achieve a full correction, and the number of strut changes and/or adjustments to achieve a full correction. See Figure 1 for the three frames that have been recently introduced in clinical practice, i.e. TL-HEX, Max Frame, and Orthex.

There is a very wide range of possible combinations of bone deformities and it is impossible to test for all kinds of deformities. Therefore, we planned to test the most common deformities that we face in clinical practice. These include (1) valgus deformity of the distal femur; (2) varus deformity of the proximal tibia; (3) lengthening of the above two deformities.

**Figure 2:**
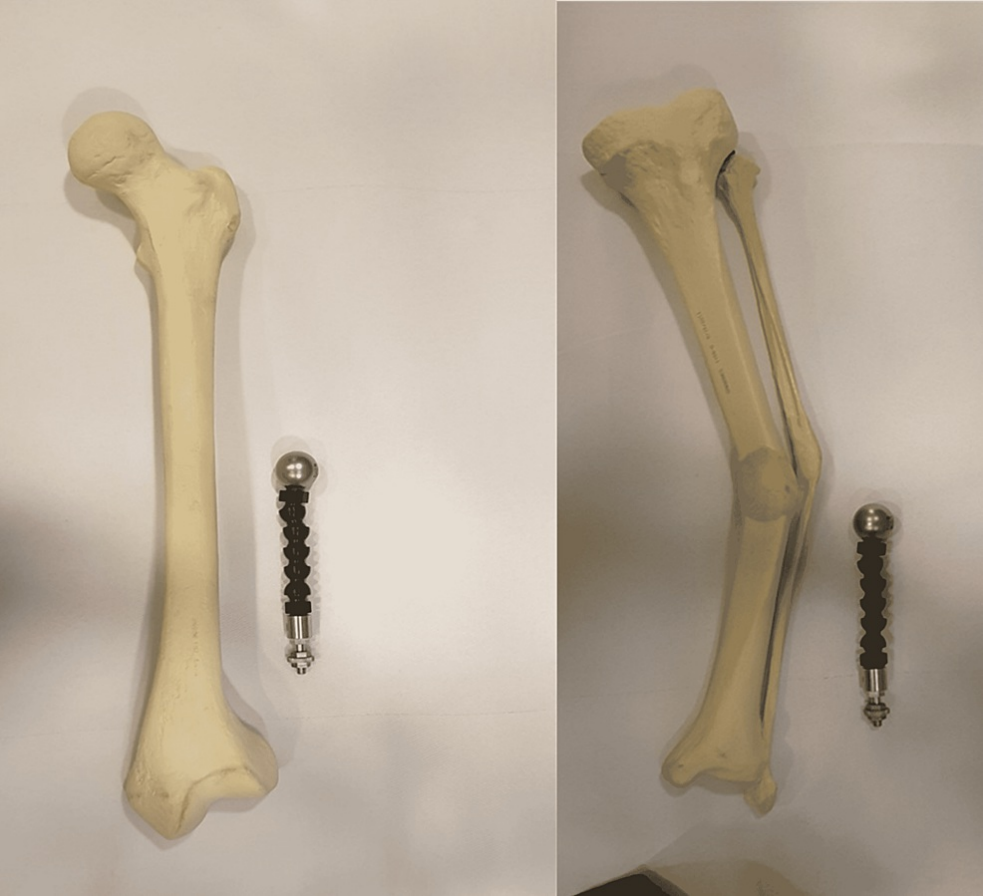
Two models of Sawbones have been used The left image is a left femur with distal valgus deformity; the right image is a left tibia with shaft varus deformity

**Figure 3:**
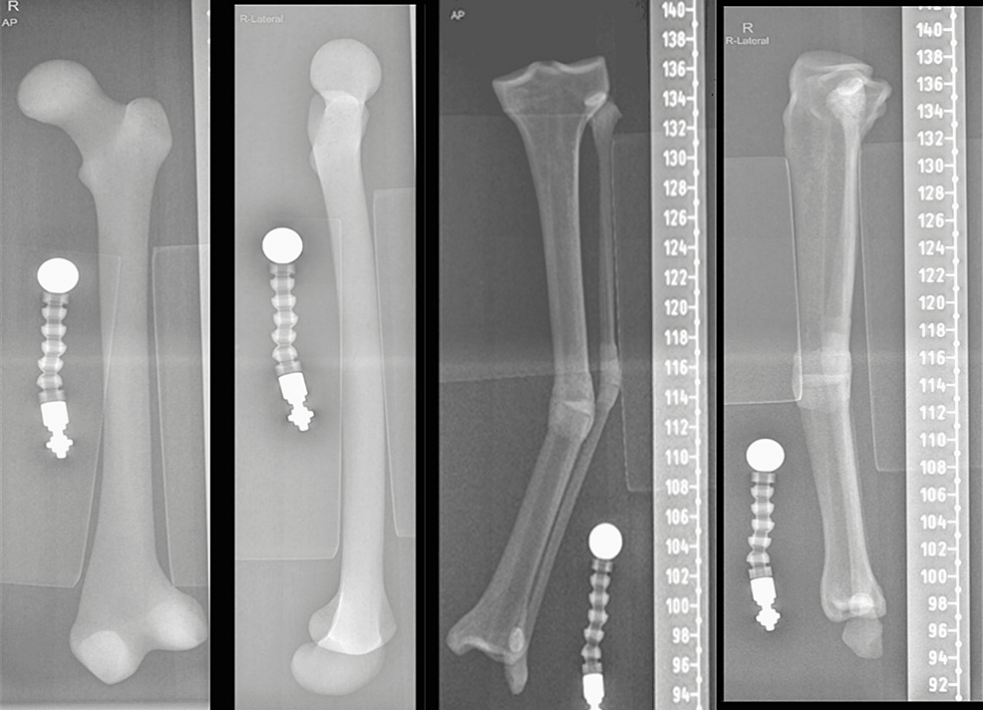
Plain radiographs of the Sawbones in Figure 2 using calibrating balls

Two models of sawbones have been used (Figures 2-3, respectively): a femur with 30° of valgus deformity of the lower end and a tibia with 30° of varus deformity in the middle of the shaft. The experiment was conducted at Al Jalila Children’s Specialty Hospital.

**Figure 4:**
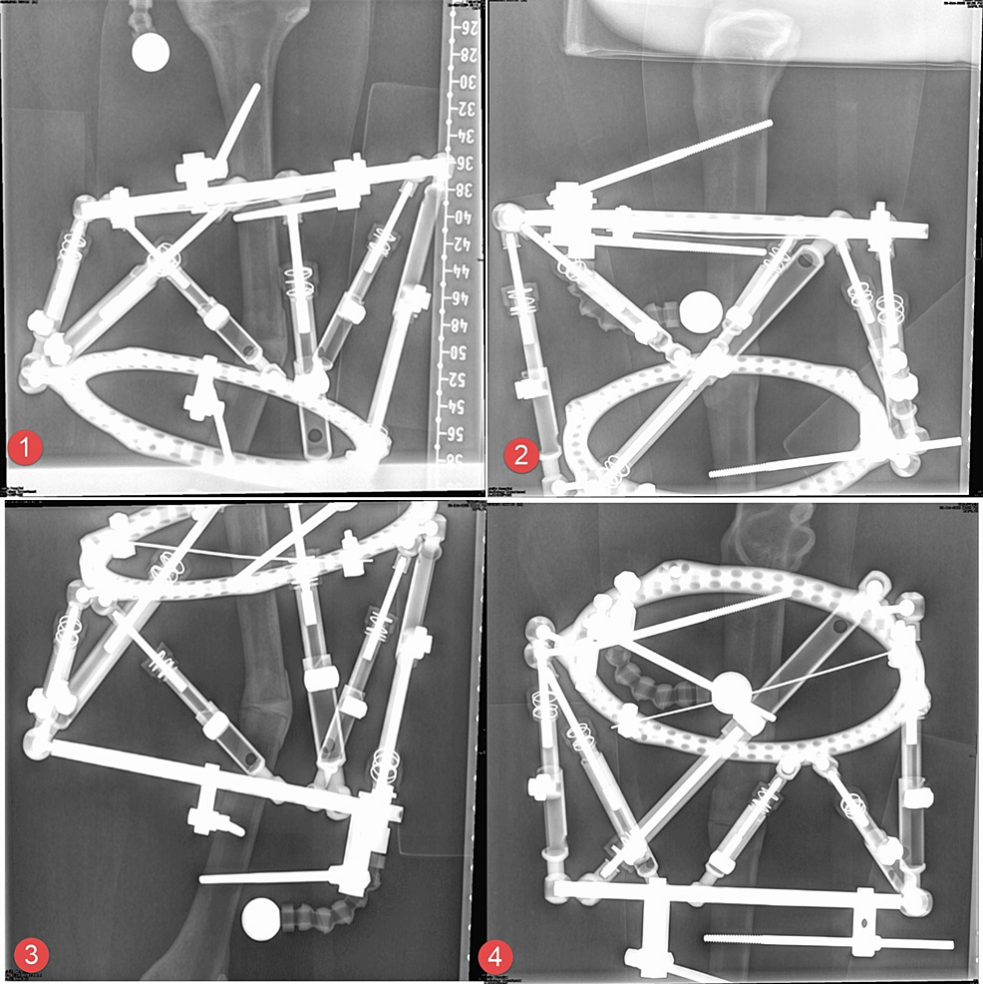
Orthogonal radiographs that are used for deformity quantification and planning

Each frame was applied to the deformed bone in the standard way that we apply to normal bone. The rings are applied orthogonally (perpendicular) to the corresponding bony segments in the sagittal and coronal planes. This was initially performed using a half pin and a polyaxial clamp; then the ring was adjusted to be orthogonal to the attached bony segment and the clamp is tightened. Two more half pins are inserted on either side of the ring to ensure stability. A proximal and distal orthogonal X-ray views were taken in the sagittal and coronal planes with a calibration ball as close to the bone as possible. Foam wedges were used to ensure the proper position of the bones in relation to the X-ray beams. Orthogonal views refer to the fact that the X-ray beam is centered on the proximal or distal ring so that it appears as a single line rather than a ring (Figure 4).

**Figure 5:**
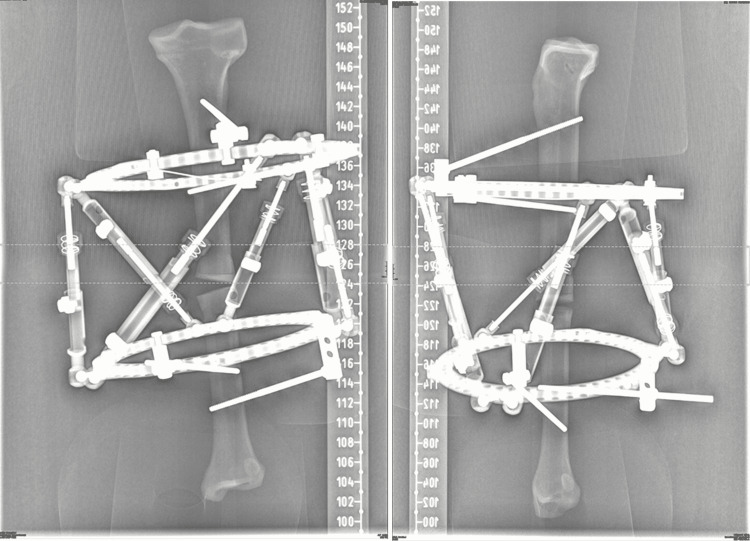
Post-correction radiograph of the tibia as an example

Deformity analysis using the relevant frame’s software was conducted and compared. The rotational deformity was assessed and entered clinically. Osteotomy of the bone at the site that was suggested by the software was then performed using an oscillating saw. The deformity correction schedules as suggested by the frame software were executed to correct the deformity for the tibia and femur using a variety of proximal and distal referencing methods. Plain X-rays were repeated until complete correction was achieved (Figure 5).

Any residual deformity was recorded and compared. Further re-programmings to achieve perfection were recorded as well. Residual deformity after initial correction and the number of re-programmings were compared among these three frames. The number of struts changes and re-adjustments was also recorded and compared. The least residual deformity, re-programming and strut changes, and re-adjustment are the favorable outcomes. Each correction was repeated eight times to minimize bias. Deviation of more than 5 degrees or 5 mm from normality was considered significant [4,11-12].

Data were checked for normality using the Shapiro-Wilk/Kolmogorov-Smirnov test as appropriate. Categorical variables were cross-tabulated and statistically tested using the chi-square or exact Fisher test depending on the number of variables. Deformity measurements whether angles or distance were recoded as continuous variables. The analysis of variance (ANOVA) or Kruskal-Wallis H test was used to compare the difference among the three frames depending on the normality of the data with the former being used for normally distributed data and the latter for non-normal data. If the difference was significant, post hoc tests were used to identify which frame(s) caused the observed difference. A P-value of less than 0.05 was considered significant in all statistical analyses. For skewed data, the validity of estimates was also checked using bootstrap techniques [13].

## Results

Accuracy of the deformity analysis

The TL-HEX and Orthex frames use their integrated web-based software tools to analyze deformity. However, TSF does not have integrated deformity analysis software. Most surgeons, including the senior authors, use tools that were developed by TraumaCAD (BrainLab, Munich, Germany) specifically for TSF. All the Sawbones models had a 30-degree actual coronal angulation. Therefore, these are not normally distributed since the deformity is pre-set at 30 degrees. However, the software estimates of these deformities were tested for normality using Kolmogorov-Smirnova Shapiro-Wilk and found data to be normally distributed (P=0.2 and 0.338), respectively. Our findings are summarised in Table 1. The estimated means of the 30-degree bone deformities were 25.35°, 25.6°, and 29.87° by the Orthex, TSF (Trauma CAD), and TL-HEX software, respectively. One-way ANOVA showed a significant difference in the findings (P-value 0.014). Post-hoc analyses using three different statistical tests (Tuckey, Least Significant difference, and Bonferroni) showed that the TL-HEX was significantly different from the TSF (Trauma CAD) and Orthex software in estimating the deformity magnitude with TL-HEX is closer to the true value (30°). It is also of note that the difference is under 5 degrees. Although it was statistically significant, it may not be so clinically.

**Table 1:** The accuracy of the deformity analysis

Items	Orthex	TSF	TL-HEX
Mean	25.35	25.6	29.87
SD	4.6	2	2.1
Counts	8	8	8

Accuracy of the deformity correction

The purpose of correction is to bring the deformity to zero, therefore, we expect the data are not normally distributed and skewed to one side (the lowest value). The formal testing of data confirmed this anticipation. Therefore, a non-parametric test was used and supplemented by bootstrap testing. The median residual deformity after the first correction was 1, 2.5, and 3° using Orthex, TSF, and TL-HEX, respectively. The independent samples Kruskal-Wallis test shows that there is no significant difference between the three groups (P=0.549). A bootstrap test at 10,000 was also performed, and it is consistent with the above findings (P-value = 0.606). The residual deformity after the second programming is getting even lesser and it was even less significant. 

The frequency of struts changes required throughout the correction was not significant among the three frames using Fisher exact test (P=0.336). As stated earlier, TSF struts are not designed to be readjusted. They allow gradual but not acute lengthening or shortening during the correction; therefore, the TSF was excluded in this comparison. There was no significant difference in struts adjustment between the TL-HEX and Orthex frames.

## Discussion

Orthopedic equipment and tools are evolving as our knowledge advances. It is essential that all newly introduced equipment undergo appropriate testing before and through its usage to ensure safety and effectiveness. This also provides critical understanding to support future advancement. Our study is set to fulfill this notion.

Our study showed that the TL-HEX software was statistically more accurate than the other frames in estimating 30-degree deformity (29.87° vs 25.35° for Orthex and 25.6 for TSF). The accuracy could be due to the advantages of the TL-HEX software, being more user-friendly and accurate, or because the team has more experience with this software. It is worth noting that although the difference is statistically significant, it may not be clinically so.

All frames managed to correct the deformity accurately, with no significant difference in the magnitude of the residual deformity. Re-programming was required in only three cases (two in TL-HEX and one in Orthex), which was not statistically significant. Given the huge experience of over 20 years using the TSF, this was not a surprising finding. It is anticipated that with time, surgeons would be more precise in using TL-HEX and Orthex.

We tried to measure the time that is required to analyze, apply a frame, and correct deformity among the various frames but because of the fact that we did not have previous clinical experience with the Orthex frame, the observed timing was significantly different and it was felt that it would not be a fair comparison with the other frames, therefore, we did not pursue this.

There are several hardware and software differences among these frames such as in identifying the reference rings, adjustment for the distal referencing, the emergency bar, and the z-bars. These can bring lots of benefits to the surgeon (and consequently to the patients), but these were not the focus of our study.

One comparative study presented at the Canadian Orthopaedic Association - Annual Meeting in 2020 compared 15 patients who were treated using the TSF and 13 patients who were treated using the Orthex frame. The software prescriptions were adjusted 2.6 time/frame for the TSF and 1.2 time/frame for the Orthex group. This study did not compare residual deformity after the first correction and did not include the TL-HEX frame [14]. These factors are taken into consideration in our biomechanical study, and we found that there was no difference in the efficacy of the frames although we found the Orthex software user-friendly. The Orthex software has an advantage in distal referencing as it uses orthopedic conventions and is therefore much simpler to learn. It is really essential that physicians appreciate the importance of understanding how the frames’ software differs when distal referencing is used.

Confirming that the three frames were equally good, opens the door for better competition profiles, which is bound to bring the cost down and is likely to benefit more and more children. Surgeons can negotiate with suppliers for better prices for their patients.

To the best of our knowledge, this is the first study that compared these three frames in terms of accuracy and strut changes. It is not sponsored by any company, negating any potential conflicts of interest. To keep the data clean and comparable, we reduced the infinite number of deformities to two only, which are by far the most commonly encountered deformities in clinical practice.

It was regrettable that we could not test the MAX frame for several logistical issues. This could be the subject of future research. It will be interesting to see whether our findings are reproduced in real clinical practice. Further research on using these frames in correcting other deformities, including foot and ankle deformities, is underway.

## Conclusions

Bones deformity is a common orthopedic problem. Our knowledge and means to correct bone deformities are advancing. New tools are continuously being introduced in clinical practice, which is healthy. Surgeons should be open to adopting newer and, likely, better tools to help patients. However, these new tools or technologies must be tested appropriately before wide-scale usage. 

The three tested frames (TSF, TL-HEX, and Orthex) showed similar capabilities in correcting a simple 30° coronal deformity. There are some hardware and software features that are useful and would have an impact on patients' journeys. The ability of TL-HEX software in predicting the best hardware to correct a specific deformity (preoperative planning) is valuable in reducing the purchased hardware, struts adjustment, and/or exchange. The Orthex software is user-friendly and more orthopedic surgeon-intuitive. Hardware is better suited for children's small limbs and the introduction of z-bars has made the use of frames in some severe deformities in short limbs possible. However, the lack of preoperative planning software is a drawback. TSF developers have been working on updating their software, which is long overdue. Future research should explore these differences in more complex deformities as well as in clinical practice.

## Figures and Tables

**Figure 6:**
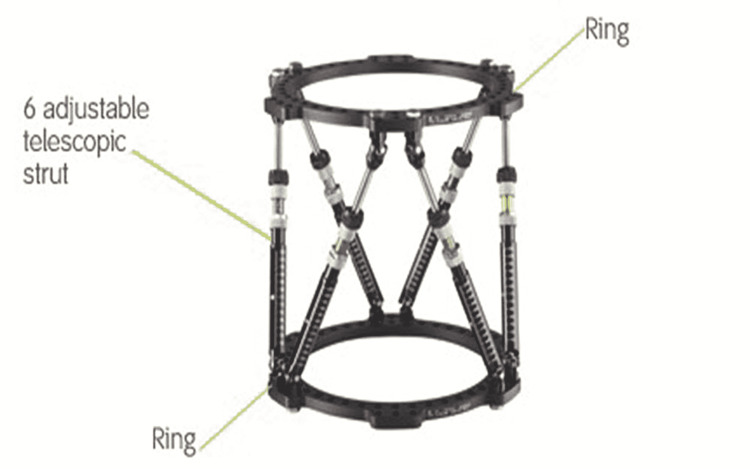
Taylor Spatial Frame

## Appendices

Appendix 1

**Table 2:** Deformity description in the three standard planes

Planes	Assessment*	Angulation	Translation
Sagittal plane (S)	X-ray (Lateral)	Procurvatum or recurvatum	Anterior or posterior
Coronal plane (C)	X-ray (AP)	Varus or valgus	Medial or lateral
Axial (A)	Clinical or CT scan	Internal or external	Short or long
* While an X-ray or CT scan is the method used in clinical practice, almost all deformity correction courses use a photograph that is taken by a camera (commonly phone cameras).			

Appendix 2

Appendix 3

**Table 3:** Some important differences between the four frames

Items	TSF	TL-HEX	Orthex	MAX
Integrated deformity analysis Software	No	Yes	Yes	Yes
Integrated mounting parameters calculation	No	Yes	Yes	Yes
Methods of calculating mounting parameters	Using the trauma CAD TSF interface, a ring, and osteotomy on AP and lateral views. The two views are linked and cannot be unsynced	Using own integrated software. Manual adjustment is required to identify the master tab on the reference ring	Using own integrated software. Using 3 calibration balls to identify the master tab on the reference ring	Using own integrated software. But use small balls on each strut proximally and distally to identify the master tab on the reference ring
Distal referencing	Some sort of adjustment is required	Some sort of adjustment is required	Built in the software	Some sort of adjustment is required
Strut adjustment (sliding and gradual)	No	Yes	Yes	Yes
